# Evaluation of the Correlation between the mRNA Expression Levels of *ystA* and *ymoA* Genes in *Y. enterocolitica* Strains with Different Enterotoxic Properties

**DOI:** 10.3390/pathogens10091136

**Published:** 2021-09-03

**Authors:** Agata Bancerz-Kisiel, Karolina Lipczyńska-Ilczuk

**Affiliations:** Department of Epizootiology, Faculty of Veterinary Medicine, University of Warmia and Mazury, Oczapowskiego 2 Str., 10-719 Olsztyn, Poland; karolina.lipczynska@uwm.edu.pl

**Keywords:** *Yersinia enterocolitica*, *ystA* gene, *ymoA* gene, gene expression, enterotoxin production

## Abstract

*Yersinia enterocolitica* is one of the main causative agents of human diarrhea. Pigs are a reservoir and the most common source of infection for humans. The aim of this study was to analyze the expression of *ystA* and *ymoA* genes in *Y. enterocolitica* strains with different enterotoxic properties, isolated from humans and pigs. The experiment involved two groups of *Y. enterocolitica* strains producing and not producing enterotoxin YstA, which were isolated from humans and pigs. All strains were *ystA-* and *ymoA*-positive. The expression of *ystA* and *ymoA* genes was analyzed by quantitative real-time PCR (qPCR). The relative expression level of the *ystA* gene was significantly higher than the expression level of the *ymoA* gene in *Y. enterocolitica* strains isolated from humans with clinical symptoms of yersiniosis. In other strains, a significant decrease in *ystA* gene transcription was observed, and the relative expression level of the *ymoA* gene was significantly higher than the expression level of the *ystA* gene. Statistically significant differences were not observed in either group of strains isolated from pigs. The results of our study revealed a correlation between mRNA expression levels of *ystA* and *ymoA* genes in *Y. enterocolitica* strains isolated from humans.

## 1. Introduction

The genus *Yersinia* consists of many species, of which *Y. pestis, Y. enterocolitica*, and *Y. pseudotuberculosis* are pathogenic for humans and animals [1]. Yersiniosis caused by *Y. enterocolitica* is an important foodborne zoonosis with growing epidemiological significance [2]. Each year, the European Food Safety Authority (EFSA) classifies yersniosis as one of the most important zoonotic diseases of the digestive tract. In humans, clinical symptoms of yersiniosis include mainly digestive disorders with acute diarrhea bowel inflammation and fever [3], while animals are usually asymptomatic carriers [4]. Six biotypes (1A, 1B, 2, 3, 4, 5) of *Y. enterocolitica* have been identified to date, including the non-pathogenic biotype 1A, weakly pathogenic biotypes 2–5, and the highly pathogenic biotype 1B [5]. *Yersinia enterocolitica* strains have been also divided into more than 70 serotypes based on the structure of the somatic antigen O [6]. Pigs are the main reservoir of *Y. enterocolitica* and a source of infection for humans. The presence of correlations between strains isolated from healthy pigs and humans with yersiniosis has been frequently confirmed by other researchers [7,8,9,10]. Undercooked pork is regarded as the main source of infection, but cross-contamination is also possible. The pathogenicity of *Y. enterocolitica* strains is determined mainly by the presence of genes referred to as virulence factors. The proteins encoded by these genes enable bacteria to penetrate sensitive organisms, colonise the digestive tract, evade the immune response, and grow under unfavorable conditions.

Virulence factors include *yst* genes that encode the production of *Yersinia* stable toxins (Yst). Yst play a significant role in the etiology of diarrhea that accompanies the disease and is one of the key virulence factors of *Y. enterocolitica*. Two main groups of *Y. enterocolitica* enterotoxins have been identified: enterotoxin YstI which includes variants YstA, YstB, and YstC, and enterotoxin YstII whose mechanism of action probably differs from that of YstI [11]. The best-known *Y. enterocolitica* enterotoxin, YstA, is a 30-amino-acid peptide whose mechanism of action is based on the activation of guanylate cyclase. This mechanism of action is highly similar to that found in heat-stable enterotoxin type I (STI) produced by *Escherichia coli,* and it is responsible for an increase in cyclic guanosine monophosphate (cGMP) levels in intestinal epithelial cells and the extracellular accumulation of liquid [12,13]. Additionally, the C-terminal 13 amino acid region of YstA corresponds to a strongly conserved sequence that is characteristic of all thermostable toxins produced by enterotoxigenic *E. coli* [14]. The opponents of the hypothesis postulating that YstA is the main cause of diarrhea during yersiniosis have pointed out that YstA is not produced at temperatures higher than 30 °C. They have argued that enterotoxin YstA is unlikely to induce diarrhea since the temperature in the intestines approximates 37 °C. However, Mikulskis et al. [15] demonstrated that *ystA* transcription can be induced at 37 °C when the pH of the culture is similar to that found in the ileum at pH 7.5. In the above conditions, YstA production was identical to that noted at temperatures below 30 °C. Enterotoxins YstB and YstC are produced by *Y. enterocolitica* strains belonging to biotype 1A. These strains are generally considered non-pathogenic, but recent research indicates that they could play a role in diarrhea induction [16].

YstA is encoded by the *ystA* gene, but not all *ystA-*positive strains produce enterotoxin. Enterotoxin-producing capabilities have been attributed to the *ymoA* gene which encodes the production of the Yersinia modulator (YmoA) protein. YmoA belongs to the family of nucleoid-associated proteins, and its sequence shows 82% identity with the regulator of high haemolysin activity (Hha) proteins in *E. coli* and *Salmonella* [17]. YmoA influences DNA supercoiling and forms heterodimers with histone-like nucleoid structuring (H-NS) proteins [18,19,20,21]. H-NS play important roles as structural proteins and gene expression modulators [22,23]. Peruzy et al. [24] recently demonstrated that 161 *Y. enterocolitica* strains of different origin, tested for the presence of the *ymoA* gene, were positive. The above observation implies that the expression of *ymoA* should be examined to evaluate its ability to modulate other genes. The expression of the genes responsible for the pathogenicity of *Y. enterocolitica* has been broadly investigated. Research results indicate that YmoA is one of the main modulators of gene expression in response to environmental factors [25,26] and that it participates in the negative regulation of virulence gene transcription [27,28]. Researchers have suggested that *ymoA* inhibits the expression of the *invA* gene and participates in VirF regulation [28] and temperature-dependent production of *Yersinia* outer proteins (Yops) and *Yersinia* adhesin (YadA)[27]

The possible influence of *ymoA* on *yst* genes was first postulated by Cornelis et al. [27] who suggested that a *ymoA* mutation unblocks the silencing of the *yst* gene and stimulates enterotoxin production. However, the results of our previous study [29] show that two-point mutations in the nucleotide sequence of the *ymoA* gene, which were detected with the use of the high-resolution melting (HRM) method, did not influence the enterotoxic properties of the examined strains. In 1994, Mikulskis et al. [15] presented the mechanism modifying the expression of *yst* to a silent state. According to the cited authors, gene silencing was caused by modifications in the status of bacterial host factors, and YmoA participated in both *yst* silencing and temperature regulation. YmoA was then identified as one of the factors necessary for the growth-phase regulation of *yst,* but physicochemical parameters—such as temperature, osmolarity, and pH—also play an important role in *yst* transcription. In 1998, Grant et al. [30] also suggested that the lack of enterotoxic properties in selected *Y. enterocolitica* strains could result from the inhibitory influence of the *ymoA* gene on *ystA* gene expression in in vitro cultures. To date, this possibility has been investigated only by Starke and Fuchs [31] who identified YmoA as a silencing factor for all toxic complex (*tc*) genes in *Y. enterocolitica* strain W22703 (biotype 2, serotype O:9).

The purpose of the study was to analyze mRNA expression levels of *ystA* and *ymoA* genes in *Y. enterocolitica* strains with different enterotoxic properties, isolated from humans and pigs.

## 2. Results

To understand in vitro expression and the possible role of the *ymoA* gene during diarrhea induction, we monitored in vivo the expression of *ystA* and *ymoA* genes in *Y. enterocolitica* strains with known enterotoxic properties. All examined strains were *ystA-*, and *ymoA*-positive, regardless of their ability to produce enterotoxins *in vivo*. *Yersinia enterocolitica* strains isolated from humans with clinical signs of yersiniosis and *Y. enterocolitica* strains isolated from pigs and capable of producing enterotoxin YstA in the suckling mouse bioassay were used in this experiment. *Yersinia enterocolitica* strains isolated from infected humans with an unknown clinical diagnosis and *Y. enterocolitica* strains isolated from pigs and not producing the enterotoxin in the suckling mice bioassay were used in the comparison.

**Figure 1:**
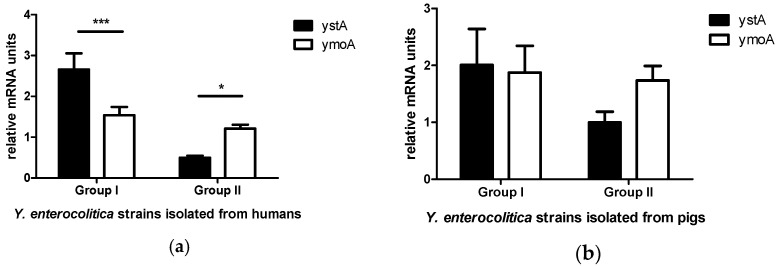
Expression of *ystA* and *ymoA* mRNA in *Y. enterocolitica* strains with different enterotoxic properties, isolated from humans and pigs. The results of real-time PCR for *ystA* and *ymoA* genes were normalized against the expression of *gapA* and *polA* genes. Data are expressed as means ± SEM, and asterisks indicate differences between groups (* *p <* 0.05, *** *p <* 0.001). (**a**) *Y. enterocolitica* strains isolated from humans; Group I consists of strains isolated from humans with clinical signs of yersiniosis; Group II consists of strains isolated from humans with an unknown clinical diagnosis (**b**) *Y. enterocolitica* strains isolated from pigs; Group I consists of strains capable of producing YstA enterotoxin in the suckling mouse bioassay; Group II consists of strains unable to produce enterotoxin.

The relative expression of the *ystA* gene was significantly higher (*p <* 0.001) than the expression of the *ymoA* gene in *Y. enterocolitica* strains isolated from humans with clinical signs of yersiniosis (Group I). The opposite was observed in the group of *Y. enterocolitica* strains isolated from humans with an unknown clinical diagnosis (Group II)—a significant decrease in *ystA* gene transcription was noted in all strains, and the relative expression of the *ymoA* gene was significantly higher (*p <* 0.05) than the expression of the *ystA* gene (Figure 1a). Therefore, a correlation was found between the relative expression of *ystA* and *ymoA* mRNA.

Statistically significant differences were not observed in either group of strains isolated from pigs. Significant differences in the relative expression levels of *ystA* and *ymoA* genes were not noted in *Y. enterocolitica* strains isolated from pigs and capable of producing enterotoxin YstA in the suckling mouse bioassay (Group I). In the group of *Y. enterocolitica* strains isolated from pigs and unable to produce enterotoxin YstA (Group II), the relative expression of the *ymoA* gene was higher than the expression of the *ystA* gene, but the observed differences were not statistically significant (Figure 1b). Therefore, unlike in humans, no correlation was found between the relative expression of *ystA* and *ymoA* mRNA.

**Figure 2:**
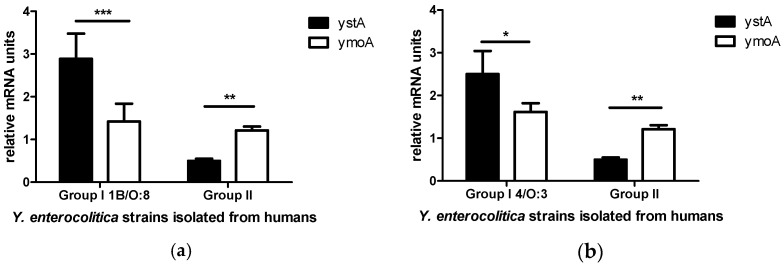
Expression of *ystA* and *ymoA* mRNA in *Y. enterocolitica* strains with different bioserotypes, isolated from humans. The results of real-time PCR for *ystA* and *ymoA* genes were normalized against the expression of *gapA* and *polA* genes. Data are expressed as means ± SEM, and asterisks indicate differences between groups (* *p <* 0.05, ** *p <* 0.01, *** *p <* 0.001). (**a**) Group I consists of *Y. enterocolitica* strains belonging to bioserotype 1B/O:8, isolated from humans with clinical signs of yersiniosis; Group II consists of strains isolated from humans with an unknown clinical diagnosis; (**b**) Group I consists of *Y. enterocolitica* strains belonging to bioserotype 4/O:3, isolated from humans with clinical yersiniosis; Group II consists of strains isolated from humans with an unknown clinical diagnosis.

The results of the statistical analysis of *ystA* and *ymoA* mRNA levels in *Y. enterocolitica* strains isolated from humans with clinical signs of yersiniosis revealed minor differences between bioserotypes. It appears that the more pathogenic the strain, the higher the expression of *ystA* is compared to *ymoA*. The significance of the observed differences was determined at *p <* 0.001 in *Y. enterocolitica* strains belonging to the highly pathogenic bioserotype 1B/O:8 (Figure 2a) and at *p <* 0.05 in *Y. enterocolitica* strains belonging to bioserotype 4/O:3 (Figure 2b). Since highly pathogenic strains of bioserotype 1B/O:8 were not isolated from humans without clinical yersiniosis, Group II strains of *Y. enterocolitica* belonging only to bioserotype 4/O:3 were used for comparison. The significance of the observed differences in *Y. enterocolitica* strains isolated from humans with an unknown clinical diagnosis (Group II) was determined at *p <* 0.01.

## 3. Discussion

It has been long suggested that YmoA is an important determinant of the production of enterotoxin Yst by *Y. enterocolitica* strains [15,27,30]. Research aiming to confirm or rule out the above hypothesis has not been undertaken since the above observation had been made. However, YmoA has been confirmed as a negative regulator of the transcription of other virulence markers, such as *inv* which encodes invasin—the essential factor of internalization, responsible for the transport of *Y. enterocolitica* across M cells [27,28]. YmoA was also shown to participate in the production of Yops and YadA which is dependent on temperature [28]. More recently, Böhme et al. [17] described YmoA as a thermo-sensitive virulence modulator protein which optimizes thermoreception and fine-tunes virulence gene expression during infection. Although the role of YmoA in the regulation of various virulence factors of *Y. enterocolitica* has been proven many times, there are still no detailed reports on their influence on the enterotoxins production. To better understand the regulatory factors that contribute to enterotoxin production by *Y. enterocolitica*, we examined the molecular mechanism that switches *ystA* expression to a silent state. Our recent study revealed that two-point mutations in the coding region of the *ymoA* gene nucleotide sequence do not affect the enterotoxic properties of the examined strains [29]. Our findings did not confirm the postulated influence of *ymoA* mutations on *ystA* gene silencing [15,27]. However, analyses of genes encoding H-NS proteins in *Yersinia* spp. are hampered by the fact that their mutations are harmful for cells [32,33]. Our study was prompted by the above observation as well as the hypothesis postulating that decreased expression of the *ystA* gene in in vitro cultures could be responsible for the absence of enterotoxic properties in selected *Y. enterocolitica* strains.

The results of this study broaden the knowledge about the interactions between *ystA* and *ymoA*, including their involvement in the pathogenicity of *Y. enterocolitica,* and the spectrum of virulence genes that are controlled by YmoA. We observed a significant reduction in the mRNA expression of the *ystA* gene in strains isolated from humans with an unknown clinical diagnosis. The relative expression level of the *ymoA* gene was significantly higher than the expression level of the *ystA* gene. In patients with clinical signs of yersiniosis, the relative expression of the *ymoA* gene was significantly lower than the expression of the *ystA* gene. The above was particularly evident in *Y. enterocolitica* strains belonging to the highly pathogenic bioserotype 1B/O:8 which is responsible for the most severe cases of the disease. Differences were also observed in the mRNA expression of the *ystA* gene in *Y. enterocolitica* strains isolated from humans with symptoms yersiniosis and belonging to bioserotype 4/O:3, but they were less significant than those noted in bioserotype 1B/O:8 strains. The above could suggest that the more pathogenic the *Y. enterocolitica* strain, the higher the expression of *ystA* compared to *ymoA*. This observation implies that the more pathogenic a strain, the higher the number of virulence genes that are regulated by YmoA.

According to our knowledge, this study is the second research attempt to investigate the influence of YmoA on the production of enterotoxins by *Y. enterocolitica*. The first attempt had been made by Starke and Fuchs [31] who demonstrated that YmoA silenced all *tc* genes of *Y. enterocolitica* strain W22703 (biotype 2, serotype O:9). The above authors relied on transcriptional fusions between the promoter and the luciferase reporter to determine that the deletion of *ymoA* increased the transcription of *tcaR1*, *tcaR2*, *tcaA*, *tcaB*, *tcaC*, *tccC1*, and *tccC2* at a temperature of 15 °C and 37 °C. They also observed that, at low temperatures, the amount of thermostable YmoA in cells was not reduced, but the repressor was less functional. In the cited study, the addition of episomal *ymoA* considerably reduced *tc* gene expression, thus confirming the inhibitory influence of YmoA on the production of insecticidal proteins. According to Starke and Fuchs [31], YmoA facilitates H-NS binding to *tc* promoters by creating a compound with this nucleoid-associated protein. The resulting compound not only binds to the upstream regions of all *tc* genes, but also to intragenic sites of *tcaA* and *tcaB;* therefore, it plays a significant role by controlling the expression of both genes. According to Starke and Fuchs [31], only *tcaA* and *tcaB* encode toxin A subunits. These observations are in line with our findings which demonstrated a correlation between the mRNA expression of *ystA* and *ymoA* genes in *Y. enterocolitica* strains isolated from humans.

No such differences were found in *Y. enterocolitica* strains isolated from pigs. Interestingly, a decrease in the mRNA expression of the *ymoA* gene was not observed in *Y. enterocolitica* strains isolated from pigs and producing enterotoxin YstA in the suckling mouse bioassay. The above could be attributed to the fact that only 13 such strains were analyzed in the present study. Perhaps a higher number of strains with a proven ability to produce YstA enterotoxin could produce statistically significant results. We also observed that the mRNA expression of the *ymoA* gene was higher than the mRNA expression of the *ystA* gene in the group of *Y. enterocolitica* strains which were isolated from pigs and not able to produce enterotoxins, but the noted differences were still not statistically significant. The above could be explained by the fact that pigs are very often asymptomatic carriers and that host-specific factors are involved in the production of virulence markers. The presence of factors that cooperate with YmoA in *Y. enterocolitica* strains isolated from humans cannot be ruled out.

## 4. Materials and Methods

### 4.1. Materials

This study was performed retrospectively based only on bacterial strains, and it did not require ethical approval. *Yersinia enterocolitica* strains were previously isolated from samples routinely submitted to diagnostic laboratories and obtained from infected humans. *Yersinia enterocolitica* strains isolated from pigs were obtained from a previous study [29]. The experimental material consisted of 74 *Y. enterocolitica* strains isolated from humans and 51 *Y. enterocolitica* strains isolated from pigs. In this study, only *Y. enterocolitica* strains from infected humans and animals (asymptomatic carriers) were analyzed

### 4.2. Yersinia enterocolitica Strains Isolated from Humans

Group I was composed of 34 *Y. enterocolitica* strains isolated from humans with clinical signs of yersiniosis (anonymous data from laboratories). Group II consisted of 40 *Y. enterocolitica* strains isolated from humans with an unknown clinical diagnosis. All *Y. enterocolitica* strains had been previously biotyped, serotyped, and molecularly examined (*ystA, ystB, ystC, ymoA*). Primer sequences and PCR conditions were described previously [14]. Group I consisted of 13 *Y. enterocolitica* strains belonging to bioserotype 1B/O:8 and 21 strains belonging to bioserotype 4/O:3 that have been rarely noted in Poland. Group II consisted of *Y. enterocolitica* strains belonging only to bioserotype 4/O:3 because highly pathogenic bioserotype 1B/O:8 strains have never been isolated from humans without clinical yersiniosis. All *Y. enterocolitica* strains used in this study were *ystA-* and *ymoA*-positive.

### 4.3. Yersinia enterocolitica Strains Isolated from Pigs

Fifty-one *Y. enterocolitica* strains isolated from fattening pigs without clinical signs of yersiniosis were examined. The enterotoxic properties of these strains were previously determined in the suckling mouse bioassay [14]. Enterotoxin production was evaluated by measuring the ratio of intestinal mass to the remaining body mass in three examined sucklings. According to Gianella [34], a ratio of ≤ 0.074 indicates a negative result, a ratio of 0.075–0.082 denotes a doubtful result, and a ratio of ≥ 0.083 represents a positive result. In this study, 13 *Y. enterocolitica* strains producing enterotoxin YstA in the suckling mouse bioassay formed Group I, and 38 *Y. enterocolitica* strains not producing enterotoxins in the suckling mouse bioassay formed Group II. All examined strains belonged to bioserotype 4/O:3 and were *ystA-* and *ymoA*-positive.

### 4.4. RNA Preparation and Reverse Transcription

Bacteria were grown in tryptic soy broth (TSB) (pH 7.3)at 28 °C, and the inoculated medium was incubated with shaking (250 rpm.) for 24 h. The cells were harvested by centrifugation in the Eppendorf Centrifuge 5804 R for 5 min at a speed of 3100× *g*, and the supernatant was discarded. Total RNA was extracted with the use of cell pellets containing 1 × 10^7^ cells and RLT Buffer in the RNeasy Protect Bacteria Mini Kit (Qiagen, Hilden, Germany). This kit includes the RNAprotect Bacteria Reagent for stabilizing RNA in bacterial samples and RNeasy spin columns for purifying up to 100 µg of high-quality RNA using the silica-membrane technology. The RNA extraction procedure was conducted according to the manufacturer’s instructions. RNA integrity was assessed by agarose gel electrophoresis. RNA concentration and quality were measured with the NanoDrop 2000 spectrophotometer (Thermo Fisher Scientific Inc., Waltham, MA, USA). An A_260_/A_280_ ratio of 2.0 (in the range of 2.06–2.13) was regarded as indicative of pure RNA. Reverse transcription (RT) to cDNA was carried out with the QuantiTect Reverse Transcription Kit (Qiagen, Hilden, Germany) according to the manufacturer’s instructions. cDNA was stored at −20 °C until further use.

### 4.5. Gene Expression Analysis Using qPCR

Selected genes were analyzed by quantitative real-time PCR (qPCR) with the Rotor-Gene6000™ real-time analyzer (Corbett Life Science, Sydney, Australia). The expression of *ystA* and *ymoA* was normalized to that of the *gapA* and *polA* reference genes which encode D-glyceraldehyde-3-phosphate dehydrogenase and produce DNA polymerase I, respectively [35]. The forward and reverse primers used in this study are shown in Table 1. Every sample for *ystA*, *ymoA*, *gapA* and*polA* mRNA analysis contained cDNA (70 ng), forward and reverse primers (final concentration of 0.7 µM/L each), and the QuantiTect SYBR Green RT-PCR master mix (Qiagen, Hilden, Germany), according to the manufacturer’s instructions. Standard curves of serial dilutions of the respective purified cDNA were used for quantification. Each PCR reaction (25 µL) was performed in a 36-well rotor under the following conditions: initial denaturation at 95 °C for 5 min, followed by 40 cycles of denaturation at 95 °C for 10 s and annealing at 52 °C for 30 s, followed by elongation at 72 °C for 45 s. Final elongation at 72 °C for 10 min was carried out for each PCR reaction. Melting curves were obtained based on a stepwise increase in the temperature ramp from 65 °C to 90 °C to ensure the amplification of a single product for each reaction. Two controls were applied in each reaction: positive control with cDNA isolated from calibrator—ACTT 23715, and negative control without cDNA. Two qPCR reactions were conducted for each *Y. enterocolitica* strain.

### 4.6. Data Analysis and Statistical Analysis

**Figure 3:**
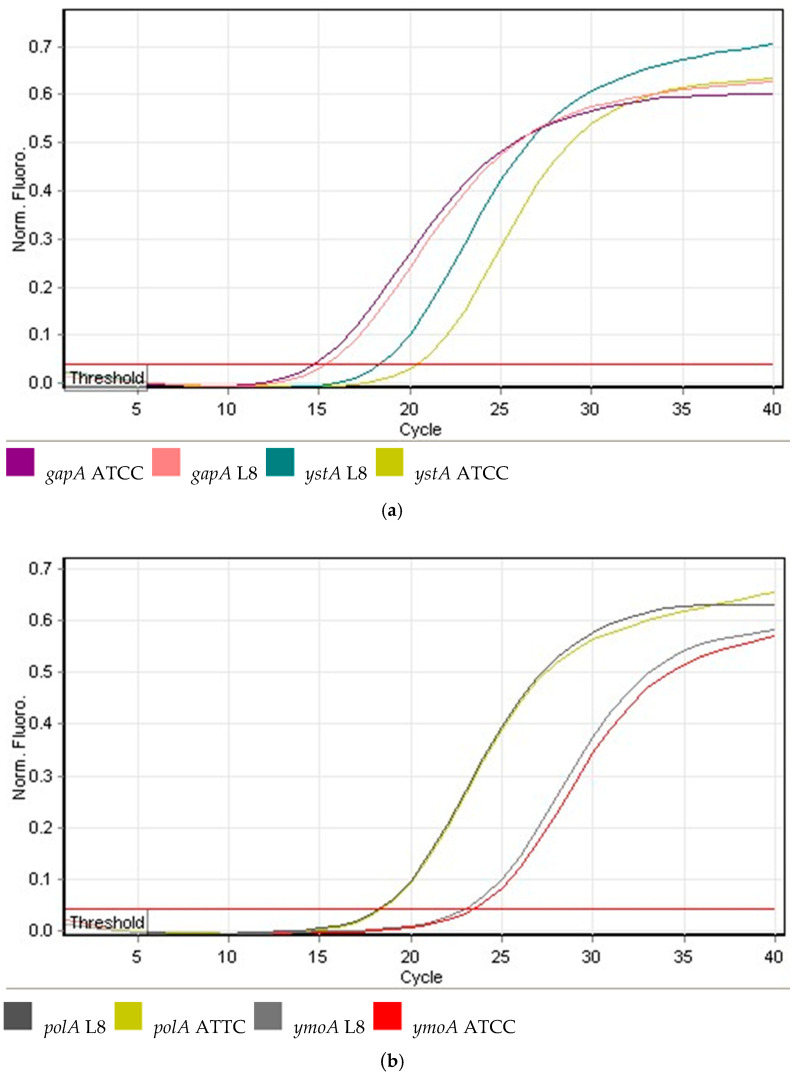
qPCR amplification results for an exemplary L8 *Y. enterocolitica* strain isolated from human and control ATCC *Y. enterocolitica* strain. The amplification curves were generated by the Rotor-Gene6000™ real-time analyzer. (**a**) The amplification curves for *ystA* and *gapA* of ACTT and L8; (**b**) The amplification curves for *ymoA* and *polA* of ACTT and L8.

Amplification curves were generated based on real-time qPCR data (example on Figure 3). The cycle threshold (CT) was calculated based on a fluorescence threshold of 0.01 and specified as the cycle in which an amplified product was first detected. ΔCT for each sample was determined using the equation Δ*CT* = *CT target gene* − *CT reference gene* to calculate the expression of each gene relative to the internal reference control. This was accomplished by modifying the original equation to the relative expression of 2−ΔCt for the samples [38,39]. All statistical analyses were performed in the Graph-Pad PRISM v. 6.0 programme (GraphPad Software, Inc., San Diego, CA, USA). The expression of *ystA* and *ymoA* mRNA in different groups of *Y. enterocolitica* strains was validated by two-way ANOVA. All numerical data were expressed as means ± SEM at a significance level of *p <* 0.05, *p <* 0.01 and *p <* 0.001.

## 5. Conclusions

The results of our study revealed a correlation between the mRNA expression of *ystA* and *ymoA* genes in *Y. enterocolitica* strains isolated from humans. No statistically significant differences were found in *Y. enterocolitica* strains isolated from pigs. It could be explained by the fact that pigs are very often asymptomatic carriers, but the presence of factors that cooperate with YmoA in *Y. enterocolitica* strains cannot be ruled out. Taking all these aspects into account, it should be stated that the presented research results should be treated as preliminary. Further analyses involving a larger number of *Y. enterocolitica* strains are needed to confirm our observation.

## Figures and Tables

**Table 1:** Sequences of the primers used in the study.

Gene	Forward Primer	Reverse Primer	Reference
*ystA*	5′GTCTTCATTTGGAGGATTCGGC3′	5′AATCACTACTGACTTCGGCTGG3′	Platt-Samoraj et al. [36]
*ymoA*	5′GACTTTTCTCAGGGGAATAC3′	5′GCTCAACGTTGTGTGTCT3′	Grant et al. [30]
*polA*	5′-GCTGGCTTGCGGATGTAGAT-3′	5′-AGCACGGCGGTCACTTCA-3′	Townsend et al. [37]
*gapA*	5′-CCATCCGTGTTACCGCAGAG-3′	5′-TCTTAGCACCAGCAGCAATGT-3′	Townsend et al. [37]

## Data Availability

The data generated and/or analyzed during the current study are available from the corresponding authors upon request.
